# Sexual risk compensation following voluntary medical male circumcision: Results from a prospective cohort study amongst human immunodeficiency virus-negative adult men in Botswana

**DOI:** 10.4102/sajhivmed.v21i1.1157

**Published:** 2020-12-14

**Authors:** Lisa P. Spees, Kathleen E. Wirth, Shreshth Mawandia, Semo Bazghina-werq, Jenny H. Ledikwe

**Affiliations:** 1 Department of Health Policy and Management, Gillings School of Global Public Health, University of North Carolina at Chapel Hill, Chapel Hill, NC, United States of America; 2 Lineberger Comprehensive Cancer Center, University of North Carolina, Chapel Hill, NC, United States of America; 3 Department of Biostatistics, Faculty of Health Sciences, Harvard T.H. Chan School of Public Health, Boston, MA, United States of America; 4 Botswana International Training and Education Center for Health (I-TECH), Gaborone, Botswana; 5 Department of Health Policy and Management, Faculty of Medicine, Botswana International Training and Education Center for Health, Gaborone, Botswana; 6 Department of Global Health, University of Washington, Seattle, WA, United States of America; 7 Independent Global Health Consultant, Washington, DC, United States of America

**Keywords:** circumcision, HIV prevention, Botswana, sexual behaviour, risk-taking, prospective studies

## Abstract

**Background:**

Circumcised men may increase sexual risk-taking following voluntary medical male circumcision (VMMC) because of decreased perceptions of risk, which may negate the beneficial impact of VMMC in preventing new human immunodeficiency virus (HIV) infections.

**Objectives:**

We evaluated changes in sexual behaviour following VMMC.

**Method:**

We conducted a prospective cohort study amongst sexually active, HIV-negative adult men undergoing VMMC in Gaborone, Botswana, during 2013–2015. Risky sexual behaviour, defined as the number of sexual partners in the previous month and ≥ 1 concurrent sexual partnerships during the previous 3 months, was assessed at baseline (prior to VMMC) and 3 months post-VMMC. Change over time was assessed by using inverse probability weighted linear and conditional logistic regression models.

**Results:**

We enrolled 523 men; 509 (97%) provided sexual behaviour information at baseline. At 3 months post-VMMC, 368 (72%) completed the follow-up questionnaire. At baseline, the mean (95% confidence interval) number of sexual partners was 1.60 (1.48, 1.65), and 111 (31% of 353 with data) men reported engaging in concurrent partnerships. At 3 months post-VMMC, 70 (23% of 311 with data) reported fewer partners and 19% had more partners. Amongst 111 men with a concurrent partnership at baseline, 52% reported none post-VMMC. Amongst the 242 (69%) without a concurrent partnership at baseline, 19% reported initiating one post-VMMC. After adjustment for loss to follow-up, risky sexual behaviour post-VMMC (measured as mean changes in a number of partners and proportion engaging in concurrency) was similar to baseline levels.

**Conclusion:**

We found no evidence of sexual risk compensation in the 3 months following VMMC.

## Introduction

Three randomised controlled trials (RCTs) from sub-Saharan Africa showed that voluntary medical male circumcision (VMMC) reduced human immunodeficiency virus (HIV) transmission from females to males by up to 60%.^1,2,3^ Mathematical models suggest that HIV incidence will decrease in circumcised men, and subsequently in women and uncircumcised men, as the uptake of VMMC increases.^4,5,6^ Botswana has the fourth highest HIV prevalence in the world, with one in five adults infected.^7^ Whilst 83% HIV-positive individuals are receiving treatment, HIV incidence remains high.^7^ In 2018, 8500 new HIV infections were diagnosed.^7^ In Botswana, it is estimated that expanding VMMC coverage would avert 4900 new HIV infections through 2030.^8^

There are concerns that circumcised men may be more likely to engage in risky sexual behaviours following surgery because of their perception of the reduced risk of HIV acquisition conferred by circumcision. This phenomenon, known as risk compensation or behaviour disinhibition, may reduce the effectiveness of VMMC in preventing new HIV infections. Although RCTs conducted in Kenya and Uganda indicated that risk compensation behaviour did not increase following circumcision,^9^ a RCT conducted in South Africa documented a higher number of sexual contacts amongst circumcised men compared with uncircumcised men in the control group.^1^ However, amongst recent cross-sectional surveys conducted in South Africa, Uganda and Kenya, there was no evidence that circumcised men’s behaviour was riskier than uncircumcised men’s behaviour.^10,11,12^

Men circumcised in non-clinical trial settings may also encounter different experiences and behave differently. However, little is known about how sexual behaviour changes in real-world settings, as there have been only two studies that examined men’s sexual behaviours before and after undergoing VMMC.^13,14^ Although the two studies, both conducted in South Africa, found minimal or no evidence of risk compensation, no studies have examined risk compensation in Botswana, where the rate of multiple and concurrent sexual partnerships is particularly high. Recently published findings from a large population-based sample found that 31% of sexually active adults in Botswana had concurrent sexual partners in the past year.^15^

We conducted a prospective cohort study amongst men aged 18–49 years undergoing VMMC in Botswana to evaluate the changes, if any, in the frequency of high-risk sexual behaviour following VMMC, including the total number and timing of sexual partnerships during the previous 3 months.

## Methods

### Study design

This clinic-based prospective cohort study was designed to assess (1) the frequency, type and severity of adverse events immediately following VMMC; (2) the prevalence and correlates of re-initiation of sexual activity and (3) changes in risky sexual behaviour following VMMC. The current analysis reports on the third primary objective. The study was conducted by the International Training and Education Center for Health (I-TECH), a collaboration with the University of Washington and the University of California, San Francisco. Recruitment and enrolment of study participants occurred before undergoing VMMC, but after individuals completed group education and individual counselling with clinic staff (including HIV testing) and provided written, informed consent for the procedure. Neither the pre-procedure activities described above nor the procedure itself was performed by study staff. The overall objective of Botswana’s National Safe Male Circumcision programme is to reach a male circumcision prevalence rate of 80% amongst 0–49-year-old HIV-negative males. A complete description of the programme, including details on the procedure itself, can be found elsewhere.^16^

### Study setting and participants

Adult men undergoing VMMC through the National Safe Male Circumcision programme were enrolled between November 2013 and April 2015 at two government-run clinics providing free circumcision services in Gaborone, the capital city of Botswana. Participant eligibility criteria included: age ranging 18–49 years, residence within 25 km of Gaborone, ever had sexual intercourse and documented HIV-negative test result. All participants provided written informed consent for participation in the study in addition to the consent obtained by clinic staff for the circumcision procedure.

### Sample size

**FIGURE 1:**
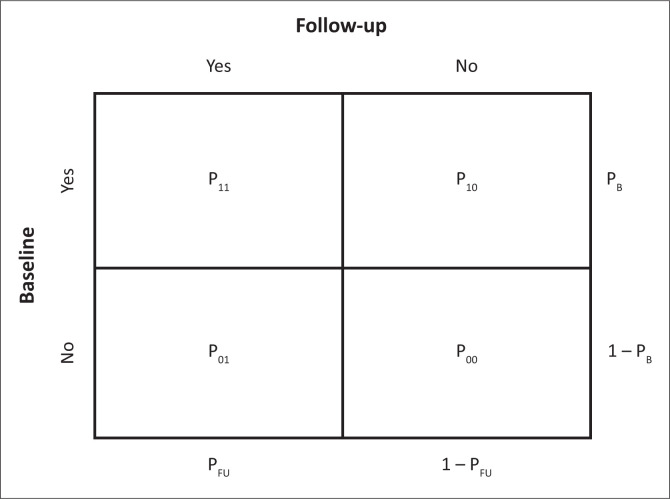
Contingency table used for calculating power based on McNemar’s test of two correlated proportions.

For study planning purposes, we computed sample size requirements (and corresponding power) based on the dichotomous outcome and engagement in concurrent sexual partnership(s) during the previous 3 months. We used McNemar’s test for two correlated proportions to determine the number of participants enrolled and the corresponding power to detect the smallest, clinically meaningful difference in the proportion of men who report engaging in a concurrent sexual partnership between baseline and 3 months post-circumcision.^17^ Figure 1 shows the summary of the proportions associated with all possible responses under this framework.

Specifically, P_B_ and P_FU_ represent the total proportion of individuals reporting high-risk sexual behaviour at baseline and follow-up, respectively; P_01_ and P_10_ represent the proportion of individuals who increase or decrease their high-risk sexual behaviour between baseline and follow-up, respectively. We explicitly chose to test a one-sided alternative that high-risk sexual behaviour increases at follow-up (as opposed to the two-sided alternative of any change) to reflect the conceptual definition of risk compensation. Thus, assuming a baseline prevalence of sexual concurrency (P_B_) of 19%,^18^ 10% were unable to undergo VMMC because of medical or other contraindications and 20% were lost to follow-up, power was estimated to be > 80% to detect a 5% increase in the proportion of men who engage in high-risk sexual behaviour at 3 months post-circumcision (relative to baseline) based on a sample size of 523 participants. Power was estimated to be 99% for a 10% increase at 3 months post-VMMC.

### Measures

In addition to the primary endpoints, the baseline questionnaire also collected information on demographics (age, current relationship status and religious affiliation), socioeconomic indicators (education, employment status, electricity in household, refrigerator in household and use of wood as cooking fuel), frequency and intensity of alcohol consumption, age at sexual initiation and history of transactional sex. We also collected information on the primary reason for getting circumcised (i.e. protecting against HIV, personal hygiene or other), whether the participant believed circumcision would have a negative, positive or no impact on the quality of sex, and administered a four-question scale developed in South Africa to assess attitudes towards condom use, monogamy and HIV risk in the context of circumcision.^19^ Lastly, we constructed two indicators evaluating the accuracy of participants’ knowledge regarding the effect of VMMC on female-to-male and male-to-female HIV transmission. Specifically, we classified men as having correct knowledge if they indicated that VMMC *partially protects* a man from getting HIV from a woman but *does not affect* a woman’s chances of getting HIV from a man.

### Data collection

Before undergoing circumcision, all study participants were asked to complete a baseline sexual behaviour questionnaire through an audio computer-assisted self-interviewing (ACASI) tool in a private office room at the study site. This interviewing technique circumvented Social Desirability bias by allowing the participant to complete the questionnaire in a standardised format without having to share their responses with study personnel directly. However, if the participant was unable or preferred not to use the ACASI tool, the study nurse was available to administer the questionnaire. The tool, available in both English and the local language, Setswana, was developed by using Questionnaire Development System (QDS^TM^) questionnaire and survey development software adapted for low literacy and computer naïve populations (Nova Research Company, Silver Spring, MD).

After circumcision, post-procedure visits at 2 days, 7 days, 6 weeks and 3 months were scheduled as outlined by the Botswana Ministry of Health (MOH) guidelines for adult VMMC. At each visit, participants completed the follow-up survey through ACASI during their clinic appointment. A follow-up visit at 12 months was planned to coincide with annual HIV testing as per standard of care in Botswana. Each study participant was provided with a wallet-size reminder card noting the date of each follow-up visit. Participants received BWP100 (approximately USD$8 at study initiation) at each post-operative visit as compensation for their time and travel costs.

The current analysis is restricted to data collected at baseline and the 3-month follow-up visit when the sexual behaviour questionnaire was repeated. Study data were collected and managed by using Research Electronic Data Capture (REDCap), a secure, web-based application designed to support data capture for research studies hosted at the Institute for Translational Health Sciences at the University of Washington.^20^

### Outcomes

The primary outcomes of the current analysis were (1) the number of sexual partners in the previous 1 month and (2) one or more concurrent sexual partnerships during the previous 3 months. In accordance with the Joint United Nations Programme on HIV/AIDS (UNAIDS) recommendations, we defined a concurrent sexual partnership as ‘overlapping sexual partnerships in which sexual intercourse with one partner occurs between two acts of intercourse with another partner’.^21^ Concurrency was then assessed by using the following three questions (based on UNAIDS recommendations for data collection), each of which was asked of participant’s three most recent sexual partners: ‘How long ago did you first have sexual intercourse with this person?’ ‘When was the last time you had sexual intercourse with this person?’ and ‘Are you still having sex with this person?’

To evaluate the change in the number of sexual partners and engagement in concurrent sexual partnerships before and after undergoing VMMC, we constructed change scores for each endpoint, which subtracted participants’ baseline response from that reported at the 3-month follow-up interview.

### Statistical analyses

For the continuous outcome, the number of sexual partners (past 1 month), we fit an intercept-only inverse-probability weighted linear regression model to estimate the mean change in the number of partners at 3 months post-VMMC (compared to baseline). For the dichotomous outcome, engagement in concurrent sexual partnerships (past 3 months), we fit an inverse-probability weighted conditional logistic regression model (stratified on the participant) to estimate the change in the proportion of participants reporting sexual concurrency at 3 months post-VMMC (compared with baseline).

Inverse probability weighting was used to adjust for potential selection bias because of non-trivial (anticipated to be 20% before study start) loss to follow-up.^22,23,24^ Inverse-probability weighting adjusts for loss to follow-up by empirically breaking the association between observed predictors (collected at baseline) and participation at follow-up, allowing for unbiased estimation in the weighted sample, provided a regression for participation is correctly specified and no unobserved correlates of non-participation and risky sexual behaviour exist. Inverse probability weights for participation at follow-up were constructed from a multivariable logistic regression model, which considered the following 24 potential covariates based on subject-matter knowledge: age, relationship status, religious affiliation, education, employment status, household assets or characteristics, reason for circumcision, correct knowledge of circumcision benefits, beliefs about circumcision, alcohol use, age at first sex, number of sexual partners (past 1 month, 1 year and lifetime) and transactional sex. We also created 117 two-way interaction terms by taking the cross-product of each demographic and socioeconomic covariates with each knowledge, belief and behavioural covariate. To build the multivariate logistic regression model required by inverse-probability weighting, we used a stepwise, forward selection procedure to identify covariates from the list of candidate predictors listed above. The entry and exit criteria were set to a *p* < 0.2. We included missing indicators for each selected variable to maximise the number of cases included in the final models and to maintain a constant sample size across analyses.

In post-hoc analyses, we sought to identify attitudes, beliefs and/or behaviours reported at baseline that may be predictive of engagement (irrespective of what the participant reported at baseline [i.e. pre-circumcision]) in high-risk sexual behaviour at 3 months post-VMMC. Specifically, we fit separate univariable- and multivariable-adjusted modified Poisson regression models (weighted by the inverse of the probability of participation at follow-up) for each of our primary outcomes (with the number of sexual partners in the past 1 month dichotomised at two or more) for each of the following covariates: alcohol consumption, reasons for circumcision, correct knowledge of circumcision benefits, beliefs about circumcision, age at first sex and transactional sex. All multivariable models were adjusted for potential confounding by the following demographic and socioeconomic covariates (all assessed at baseline): age, relationship status, religious affiliation, education, employment and household use of wood as cooking fuel.

All analyses were conducted by using SAS software version 9.4 (SAS Institute, Cary, NC).

### Ethical consideration

Ethical approvals were obtained from the Health Research and Development Committee at the Botswana Ministry of Health (MOH) (#00699) and the University of Washington Institutional Review Board (#42047).

## Results

**FIGURE 2:**
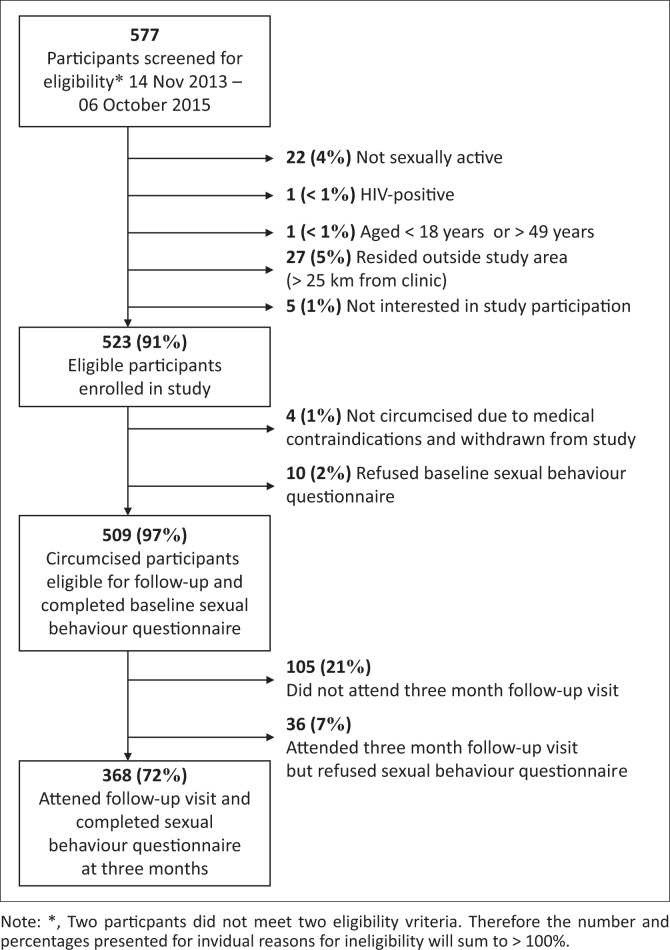
Consolidated Standards of Reporting Trials (CONSORT) diagram illustrating screening, eligibility, enrolment and follow-up of men undergoing voluntary medical male circumcision in Gaborone, Botswana, 2013–2015.

Between November 2013 and October 2015, research staff screened 577 men preparing to undergo VMMC for study participation (Figure 2). A total of 528 (92%) participants were determined to be eligible for participation and 523 (91%) subsequently enrolled. Reasons for ineligibility included not sexually active (4%), residence outside of the area (5%), HIV infection (< 1%) and age < 18 years or > 49 years (< 1%). Four individuals who met study eligibility criteria and consented to study participation were not circumcised because of medical contraindications that were identified before the procedure. Amongst the 509 circumcised participants who completed the baseline sexual behaviour questionnaire, 368 (72%) attended the follow-up visit and completed the sexual behaviour questionnaire at 3 months post-circumcision. Because of missing data on specified outcomes, 353 were included in the analysis examining the number of sexual partners in the past month, and 311 were included in the sexual concurrency analysis.

Table 1 shows the summary of the baseline characteristics of the study population according to the availability of sexual behaviour data at 3 months post-circumcision. Men who either did not complete the follow-up visit (*n* = 105) or refused the sexual behaviour questionnaire (*n* = 36) were more highly educated (odds ratio [OR]: 1.72; 95% confidence interval [CI]: 1.13–2.61) and underwent circumcision for personal hygiene reasons (OR: 1.66; 95% CI: 1.07–2.57) compared with respondents at 3 months post-VMMC. In contrast, non-respondents were less likely to live in larger households (OR: 0.59; 95% CI: 0.36–0.97). No other baseline characteristics were significantly associated with the response at 3 months post-VMMC.

**TABLE 1:** Distribution of baseline characteristics of *N* = 509 HIV-negative, sexually active adult men who underwent voluntary medical male circumcision according to the availability of sexual behaviour data at 3 months post-voluntary medical male circumcision and association of these characteristics with data availability.

Baseline characteristics (*n* with data)	Sexual behaviour data at 3 months Post-VMMC, †					
	Available (*n* = 368)		Not available (*n* = 141)		OR	95% CI
	*N*	%	*N*	%		
**Demographic**						
Age (*n* = 509) (years)						
18–24	110	30	53	38	1	ref.
25–29	123	33	50	36	0.84	0.53–1.34
30–34	76	21	22	16	0.60	0.34–1.07
35–49	59	16	16	11	0.56	0.30–1.07
Relationship status (*n* = 509)						
Single‡	71	19	26	18	1	ref.
Dating and living together	72	20	24	17	0.91	0.48–1.73
Dating but not living together	196	53	76	54	1.06	0.63–1.78
Married	29	8	15	11	1.41	0.66–3.05
Religious affiliation (*n* = 508)						
No religious affiliation	21	6	5	4	1	ref.
Any religious affiliation	346	94	136	96	1.65	0.61–4.47
**Socioeconomic**						
Highest level of education completed (*n* = 492)§						
Secondary or less	168	47	44	34	1	ref.
Higher than secondary	193	54	87	66	1.72	1.13–2.61
Employed or self-employed (*n* = 509)	263	72	96	68	0.85	0.56–1.30
Household size (*n* = 507)						
0–2	98	27	52	37	1	ref.
3–4	124	34	39	28	0.59	0.36–0.97
5–6	79	22	31	22	0.74	0.43–1.26
≥ 7	65	18	19	14	0.55	0.30–1.02
Electricity in household (*n* = 509)	314	85	121	86	1.04	0.60–1.81
Refrigerator in household (*n* = 509)	282	77	112	79	1.18	0.73–1.89
Household use of wood as cooking fuel (*n* = 509)	61	17	23	16	0.98	0.58–1.66
**Circumcision knowledge and beliefs**						
Reasons for circumcision (*n* = 509)						
HIV protection	180	49	61	43	1	ref.
Personal hygiene	98	27	55	39	1.66	1.07–2.57
Other	90	25	25	18	0.82	0.48–1.39
Knowledge of HIV risk						
Correct knowledge: VMMC partially reduces HIV risk for men (*n* = 509)	311	85	112	79	0.71	0.43–1.16
Correct knowledge: VMMC does not impact HIV risk for women (*n* = 506)	40	11	15	11	0.97	0.52–1.81
Risk compensation scale, mean (SD)¶						
Condom use is not necessary if the man is circumcised (*n* = 509)	4.35	1.00	4.28	1.26	0.94	0.79–1.12
If I am circumcised, sex is safe without a condom (*n* = 509)	4.42	0.90	4.35	1.13	0.94	0.77–1.14
Being circumcised means a man can worry less about HIV (*n* = 508)	3.79	1.46	3.91	1.42	1.06	0.93–1.22
If a man is circumcised, he can have more sexual partners (*n* = 509)	4.51	0.73	4.47	1.03	0.95	0.75–1.19
**Behavioural**						
Alcohol consumption (*n* = 509)	224	61	88	62	1.07	0.72–1.59
Age at first sexual intercourse (*n* = 501) (years)						
< 18	104	28	45	32	1	ref.
18–20	164	45	65	46	1.09	0.69–1.72
≥ 21	93	25	31	22	1.40	0.82–2.38
≥ 2 partners, past 1 month (*n* = 461)	148	44	59	47	1.11	0.74–1.68
≥ 2 partners, past 12 months (*n* = 466)	201	60	78	61	1.06	0.70–1.61
Number of partners, lifetime (*n* = 505)						
1 partner	33	9	18	13	1	ref.
2–4 partners	150	41	52	37	0.64	0.33–1.22
5–10 partners	122	33	47	34	0.71	0.36–1.37
11 or more partners	60	16	23	16	0.71	0.33–1.49
Exchanged money for sex, past 12 months (*n* = 468)	23	7	10	8	1.17	0.54–2.53

VMMC, voluntary medical male circumcision; OR, odds ratio; HIV, human immunodeficiency virus; SD, standard deviation.

† , Data are *n* (column %) unless otherwise noted. Values may not total to 100% because of rounding.

‡ , Category includes single, separated, divorced and widowed participants.

§ , Category includes participants reporting no school attendance, non-formal schooling, primary education and secondary education.

¶ , Median (25th, 75th percentile) of Likert scale (range 0–5 with 0 and 5 denoting ‘strongly disagree’ and ‘strongly agree’, respectively).

**FIGURE 3:**
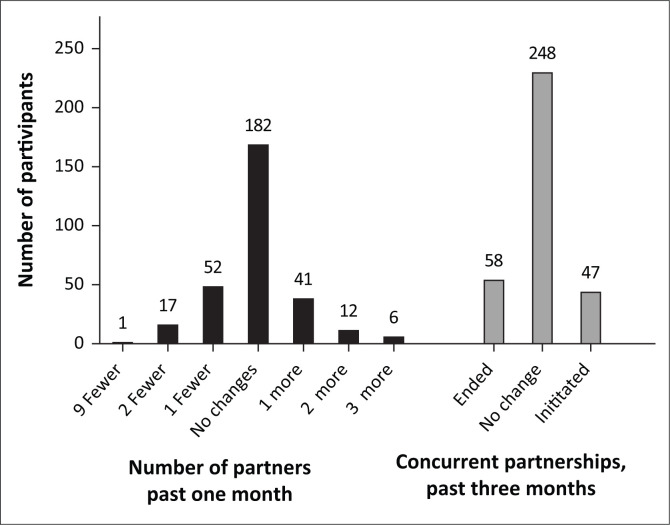
Summary of change in the number of sexual partners (past 1 month) and engagement in concurrent partnerships at 3 months amongst men undergoing voluntary medical male circumcision in Gaborone, Botswana, 2012–2015.

Figure 3 shows the summary of the change in the number of sexual partners and engagement in concurrent sexual partnerships at the 3-month follow-up visit (relative to baseline). Amongst the 368 men who attended the follow-up visit at 3 months, 57 (15%) did not provide information on the number of partners during the past 1 month at either baseline and/or follow-up. Data on engagement in concurrent sexual partnerships were available for 353 (96%) of the 368 men who attended follow-up.

At baseline, the mean (95% CI) number of sexual partners (past 1 month) was 1.60 (1.48, 1.65). Although the majority (59% of *n* = 311 with available data) of men reported the same number of partners at 3 months post-VMMC, 70 (23%) reported fewer partners and 59 (19%) had more partners (Figure 3). For concurrency (past 3 months), at baseline, 111 (31% of *n* = 353 with available data) reported engaging in one or more concurrent sexual partnerships. By 3 months post-VMMC, 58 (52% of 111) had ended the concurrent partnership. In contrast, amongst the 242 (69%) who did not report any concurrent sexual partnerships at baseline, 47 (19% of 242) had initiated a concurrent partnership at 3 months (Figure 3). After adjustment for possible selection bias because of loss to follow-up at 3 months, we found no evidence of sexual risk compensation following VMMC: mean change in number of partners was -0.04 (95% CI: -0.15, 0.08; *p* = 0.61) and the proportion engaging in concurrency was 0.04 (95% CI: -0.05, 0.14; *p* = 0.38).

Tables 2 and 3 present the results of post-hoc analyses aimed at identifying predictors of any engagement (irrespective of change from baseline or pre-circumcision) in high-risk sexual behaviour at 3 months post-VMMC. In multivariable-adjusted models (simultaneously adjusted for potential selection bias because of loss to follow-up), alcohol consumption (Table 2; risk ratio [RR]: 1.72; 95% CI: 1.19, 2.49) and transactional sex (RR: 1.77; 95% CI: 1.13, 2.77) were positively associated with reporting two or more partners (past 1 month) at follow-up. Alcohol consumption was also positively associated with concurrency at 3 months post-VMMC (Table 3; RR: 2.30; 95% CI: 1.45, 3.66). In contrast, older age at first sexual intercourse (21 years and older) was negatively associated with multiple sexual partners (Table 2; RR: 0.50; 95% CI: 0.30, 0.84) at follow-up. No other attitudinal or behavioural covariates were significantly associated with either outcome in either univariable- or multivariable-adjusted analyses.

**TABLE 2:** Univariable- and multivariable-adjusted attitudinal and behavioural predictors of multiple sexual partners (past 1 month) at 3 months amongst men undergoing voluntary medical male circumcision in Gaborone, Botswana, 2012–2015.

Attitudinal and behavioural predictors	Univariable-adjusted†			Multivariable-adjusted†,‡		
	RR	95% CI	*p*	RR	95% CI	*p*
**Reasons for circumcision**						
Personal hygiene	1	ref.	-	1	ref.	-
HIV protection	0.96	0.68–1.35	0.80	0.99	0.70–1.40	0.96
Other	0.90	0.60–1.35	0.61	0.86	0.58–1.28	0.45
**Knowledge of HIV risk**						
Correct knowledge: VMMC partially reduces HIV risk for men	1.12	0.71–1.77	0.62	1.15	0.73–1.83	0.54
Correct knowledge: VMMC does not impact HIV risk for women	1.14	0.73–1.78	0.55	1.13	0.71–1.80	0.60
**Risk compensation scale§**						
Condom use is not necessary if the man is circumcised	1.02	0.86–1.21	0.80	1.04	0.86–1.25	0.69
If I am circumcised, sex is safe without a condom	0.99	0.85–1.17	0.95	0.97	0.82–1.14	0.70
Being circumcised means a man can worry less about HIV	1.03	0.92–1.14	0.63	1.05	0.94–1.17	0.36
If a man is circumcised, he can have more sexual partners	1.10	0.87–1.39	0.42	1.08	0.86–1.37	0.49
Alcohol consumption	1.80	1.23–2.62	0.002	1.80	1.23–2.62	0.004
**Age at first sexual intercourse (years)**						
< 18	1	ref.	-	1	ref.	-
18–20	0.98	0.72–1.34	0.92	0.92	0.68–124	0.59
≥ 21	0.51	0.30–0.85	0.01	0.50	0.30–0.84	0.01
Exchanged money for sex, past 12 months	1.64	1.07–2.52	0.02	1.77	1.13–2.77	0.01

RR, risk ratio; CI, confidence intervals; HIV, human immunodeficiency virus; VMMC, voluntary medical male circumcision.

† , Estimated from a weighted modified Poisson regression model with weights constructed to adjust for selection bias because of loss to follow-up.

‡ , Estimated from a weighted modified Poisson regression model with weights constructed to adjust for selection bias because of loss to follow-up and confounding because of the following baseline covariates: age, relationship status, religious affiliation, education, employment and household use of woods as cooking fuel.

§ , Responses based on Likert scale (range 0–5 with 0 and 5 denoting ‘strongly disagree’ and ‘strongly agree’, respectively).

**TABLE 3:** Univariable- and multivariable-adjusted attitudinal and behavioural predictors of concurrent sexual partnerships (past 3 months) at 3 months amongst men undergoing voluntary medical male circumcision in Gaborone, Botswana, 2012–2015.

Attitudinal and behavioural predictors	Univariable-adjusted†			Multivariable-adjusted†,‡		
	RR	95% CI	*p*	RR	95% CI	*p*
**Reasons for circumcision**						
Personal hygiene	1	ref.	-	1	ref.	-
HIV protection	1.16	0.74–1.82	0.52	1.24	0.79–1.96	0.35
Other	0.98	0.57–1.67	0.94	0.91	0.55–1.53	0.74
**Knowledge of HIV risk**						
Correct knowledge: VMMC reduces HIV risk for men	0.99	0.58–1.68	0.97	1.05	0.59–1.86	0.87
Correct knowledge: VMMC does not impact HIV risk for women	1.23	0.71–2.10	0.46	1.16	0.66–2.01	0.61
**Risk compensation scale§**						
Condom use is not necessary if the man is circumcised	1.13	0.91–1.40	0.29	1.18	0.93–1.49	0.17
If I am circumcised, sex is safe without a condom	1.00	0.81–1.22	0.97	1.00	0.80–1.23	0.97
Being circumcised means a man can worry less about HIV	1.01	0.89–1.14	0.89	1.02	0.91–1.16	0.70
If a man is circumcised, he can have more sexual partners	1.23	0.90–1.68	0.20	1.21	0.89–1.66	0.23
Alcohol consumption	1.97	1.00–3.87	0.05	2.30	1.45–3.66	0.0004
**Age at first sexual intercourse (years)**						
< 18	1	ref.	-	1	ref.	-
18–20	1.15	0.76–1.74	0.46	1.09	0.73–1.62	0.68
≥ 21	0.59	0.32–1.06	0.08	0.71	0.38–1.34	0.29
Exchanged money for sex, past 12 months	1.57	0.87–2.82	0.14	1.62	0.99–2.63	0.05

RR, risk ratio; CI, confidence intervals; HIV, human immunodeficiency virus; VMMC, voluntary medical male circumcision.

† , Estimated from a weighted modified Poisson regression model with weights constructed to adjust for selection bias because of loss to follow-up.

‡ , Estimated from a weighted modified Poisson regression model with weights constructed to adjust for selection bias because of loss to follow-up and confounding because of the following baseline covariates: age, relationship status, religious affiliation, education, employment and household use of woods as cooking fuel.

§ , Responses based on Likert scale (range 0–5 with 0 and 5 denoting ‘strongly disagree’ and ‘strongly agree’, respectively).

## Discussion

In this prospective cohort study of adult men undergoing circumcision within an urban, public-sector clinic in Botswana, we found no evidence of sexual risk compensation in the 3 months following the procedure. Although most participants did not alter their behaviour between baseline and follow-up, amongst those whose behaviour did change, a larger proportion of men reduced, rather than increased, their engagement in risky sexual behaviour as assessed by both the total number and timing of sexual partnerships. Our overall findings are consistent with previous reports from South Africa showing little to no evidence of risk compensation following VMMC.^13,14^

Alcohol consumption was the strongest predictor of subsequent engagement in risky sexual behaviour following VMMC. Previous studies of HIV prevention interventions conducted amongst HIV-negative and HIV-positive persons in the region have yielded similar results. Data from the Sustainable East Africa Research in Community Health (SEARCH) study, a cluster-randomised test-and-treat trial conducted in rural Uganda and Kenya, identified alcohol use as a significant predictor of HIV acquisition in developing an HIV risk score.^25^ Additionally, in a subset of SEARCH participants also evaluated for tuberculosis acquisition, alcohol users were twice as likely to become infected with tuberculosis compared with non-drinkers, irrespective of HIV status or presence of an infected household contact.^26^ In Botswana, qualitative in-depth interviews conducted with sexually active young adults presenting for care for urogenital complaints cited alcohol use as contributing to inconsistent and/or incorrect condom use.^27^

Most of the existing data around risky sexual behaviour and VMMC relies on cross-sectional surveys (i.e. collected at a single point in time) in which the sexual behaviours of circumcised and uncircumcised men are compared.^10,11,12^ In contrast, we employed a pre–post intervention design in which sexual behaviour was assessed before and after participants underwent VMMC. This is important because men who choose to undergo circumcision may differ systematically from men who do not in ways that are related to risky sexual behaviour but are not easily measured. In our study, each participant served as his own control over time enabling us to control for bias resulting from fixed, systematic differences regardless of whether they are recorded by research staff. A second key strength of our results is the use of ACASI to collect sexual behaviour information. Given the sensitive and private nature of participation in sexual activities, individuals may underestimate or in some cases overestimate engagement in risky sexual behaviour. Indeed, multiple studies have shown that this type of information bias is particularly prevalent in studies on sexual behaviour, which utilise face-to-face interviewing techniques.^28,29^ Misreporting of high-risk sexual behaviour amongst men undergoing VMMC may underestimate prevalence, hindering the effectiveness and safety of circumcision programmes. To mitigate this potential form of information bias, we used ACASI, an interviewing technique that allows the participant to complete the questionnaires privately and without directly sharing their responses with study staff.

Our study is subject to limitations. Firstly, 28% of men either did not return for follow-up at 3 months post-VMMC and/or did not provide information on risky sexual behaviour post-VMMC. Adjustment for selection bias because of this sub-optimal retention relies on the assumption that, conditional on a set of observed covariates, men who did and did not return for follow-up are exchangeable, and its appropriateness depends on our ability to identify, measure and control for all covariates associated with loss to follow-up. Based on subject-matter knowledge, we considered 24 demographic, socio-economic and behavioural covariates and 117 two-way interaction terms in the multivariable model required by the inverse probability weighting procedure. However, as with any observational study, bias because of unmeasured factors is possible.

## Conclusion

Despite these limitations, our results provide reassurance that men undergoing VMMC in Botswana are unlikely to be systematically engaging in risk compensation. Such behaviour, if practised widely, has the potential to undermine the public health benefits of VMMC. Instead, we found that in the 3 months following VMMC, both the overall number and timing of sexual partners were similar to pre-circumcision levels. Botswana’s circumcision programme continues to provide HIV prevention through the circumcision procedure itself and is not being jeopardised by the perception of reduced risk of HIV acquisition.
